# Chronic Stress Alters Hippocampal Renin-Angiotensin-Aldosterone System Component Expression in an Aged Rat Model of Wolfram Syndrome

**DOI:** 10.3390/genes14040827

**Published:** 2023-03-30

**Authors:** Marite Punapart, Riin Reimets, Kadri Seppa, Silvia Kirillov, Nayana Gaur, Kattri-Liis Eskla, Toomas Jagomäe, Eero Vasar, Mario Plaas

**Affiliations:** 1 Laboratory Animal Centre, Institute of Biomedicine and Translational Medicine, University of Tartu, 14B Ravila Street, 50411 Tartu, Estonia; 2 Department of Physiology, Institute of Biomedicine and Translational Medicine, University of Tartu, 19 Ravila Street, 50411 Tartu, Estonia

**Keywords:** Wolfram syndrome, *Wfs1* knock-out, GLP1-R agonist, liraglutide, 7,8-DHF, RAAS, *Agtr2*, *Bdkrb1*

## Abstract

Biallelic mutations in the gene encoding WFS1 underlie the development of Wolfram syndrome (WS), a rare neurodegenerative disorder with no available cure. We have previously shown that *Wfs1* deficiency can impair the functioning of the renin-angiotensin-aldosterone system (RAAS). The expression of two key receptors, angiotensin II receptor type 2 (*Agtr2*) and bradykinin receptor B1 (*Bdkrb1*), was downregulated both in vitro and in vivo across multiple organs in a rat model of WS. Here, we show that the expression of key RAAS components is also dysregulated in neural tissue from aged WS rats and that these alterations are not normalized by pharmacological treatments (liraglutide (LIR), 7,8-dihydroxyflavone (7,8-DHF) or their combination). We found that the expression of angiotensin II receptor type 1a (*Agtr1a*), angiotensin II receptor type 1b (*Agtr1b*), *Agtr2* and *Bdkrb1* was significantly downregulated in the hippocampus of WS animals that experienced chronic experimental stress. Treatment-naïve WS rats displayed different gene expression patterns, underscoring the effect of prolonged experiment-induced stress. Altogether, we posit that *Wfs1* deficiency disturbs RAAS functioning under chronic stressful conditions, thereby exacerbating neurodegeneration in WS.

## 1. Introduction

Wolfram syndrome (WS; Appendix A includes a list of abbreviations used) is a rare monogenic neurodegenerative disease caused by biallelic mutations in the gene encoding the transmembrane glycoprotein Wolframin (WFS1). Disease manifestation typically begins with juvenile-onset diabetes mellitus, diabetes insipidus and loss of vision (due to optic nerve atrophy) and is often accompanied by sensorineural deafness and neuropsychiatric abnormalities, among other complications [1,2]. The incidence can vary by ethnicity, ranging from 1/770,000 in the United Kingdom to 1/68,000 in Lebanon, for instance [3,4].

*Wfs1* is broadly expressed in several tissues, with higher levels in the brain, pancreas, lungs, heart and retina [5,6,7,8]. WFS1 is primarily involved in regulating Ca^2+^ homeostasis and the endoplasmic reticulum (ER) stress response [9,10]. Additionally, *Wfs1* deficiency is associated with disruptions in mitochondrial activity, including changes in mitochondrial dynamics and degradation rate [11]. Several unfolded protein response modulators are localized in mitochondria-associated ER membranes (MAMs); these structures facilitate ER-mitochondria interactions that are critical for regulating several functions, including Ca^2+^ signaling and metabolism. MAM dysfunction can directly impact cell survival and has been implicated in various metabolic and neurodegenerative disorders. WFS1 also localizes in MAMs, and its absence in fibroblasts results in Ca^2+^ exchange disturbances and reduced ER-mitochondria contact formation in vitro [12,13].

While there are currently no curative treatments available for WS, drug-repurposing efforts have identified several promising candidates, including ER stress modulators (e.g., valproate (VPA), originally a first-choice anti-epileptic drug), chemical chaperones (e.g., sigma-1 receptor (S1R) agonists), and antidiabetics (e.g., glucagon-like peptide 1 receptor (GLP-1R) agonists). For instance, S1R agonists restored mitochondrial function and alleviated behavioral deficits in WS animal models [14]. VPA was shown to induce WFS1 expression, modulate the ER stress response and reduce apoptosis in vitro [15,16], as well as ameliorate glucose tolerance in WS mice [17]. Similarly, dantrolene (a skeletal muscle relaxant) suppressed ER stress-mediated cell death in both in vitro and in vivo WS models [18]. VPA and dantrolene are also already being explored in clinical trials (clinical trial identifiers: NCT03717909/NCT04940572 and NCT02829268, respectively; [19]). Interestingly, some drug candidates from across the neurodegenerative spectrum have also demonstrated disease-modifying potential in both in vivo and in vitro WS models. Riluzole, one of the few drugs approved for the treatment of amyotrophic lateral sclerosis (ALS), regulated aberrant glutamate transporter expression in *Wfs1*-deficient cerebral organoids, thereby restoring synapse formation and functionality. It also improved spatial memory and depressive behavior in *Wfs1* conditional knock-out mice [20]. A combination of 4-phenylbutyrate and tauroursodeoxycholic acid, also recently approved in the United States for the treatment of ALS [21], increased WFS1 levels, alleviated ER stress and inhibited cellular apoptosis in patient-derived induced pluripotent stem cells. Moreover, this combination also stimulated insulin secretion in stem cell-derived β cells and delayed the progression of diabetes in *Wfs1*-deficient mice [22]. For a comprehensive overview of potential treatment strategies for WS, interested readers may refer to [23].

Antidiabetic GLP-1R agonists in particular have shown promising results by ameliorating disease progression in both rodent models [24,25,26,27,28,29] and human patients [30,31]. More specifically, our group has shown that the GLP-1R agonist liraglutide (LIR) delays the progression of diabetes, loss of vision and neurodegeneration and improves cognitive function in a rat model of WS [24,25,26,27]. An additional trial investigating combination therapy of GLP-1 and glucose-dependent insulinotropic polypeptide receptor agonists will also be underway soon (clinical trial identifier: NCT05659368). However, the mechanisms underlying LIR’s therapeutic effects remain to be elucidated.

Additionally, we have recently shown that the renin-angiotensin-aldosterone system (RAAS) is significantly affected in *Wfs1*-deficient rats; the expression of two key RAAS receptors, angiotensin II receptor type 2 (*Agtr2*) and bradykinin receptor B1 (*Bdkrb1*)*,* was markedly downregulated both in vivo (heart and lungs) and in vitro (in primary cortical neurons). Furthermore, deficient rats had decreased aldosterone and increased bradykinin serum levels, both of which are important hormone modulators of the RAAS. Interestingly, LIR was able to modulate these levels [32], which is consistent with our previous findings that RAAS components can be pharmacologically modulated by LIR [33,34].

The RAAS regulates critical functions, including body fluid volume and blood pressure, and its dysregulation is implicated in many conditions, including cancer, diabetes and neurodegenerative disorders [35,36,37]. Importantly, in addition to the “classical” systemic RAAS, tissue-specific “micro-RAASs” have been described for several organs, including the brain and pancreas. These micro-RAASs participate in various cellular processes, including vasodilation and vasoconstriction, proliferation and regeneration and inflammatory responses [38,39,40].

Importantly, the RAAS is also associated with ER stress regulation, mitochondrial functioning and MAMs [41]. Key RAAS components are located in the mitochondria of various tissues, e.g., the adrenal glands, kidneys, liver, heart, and brain (specifically in dopaminergic neurons) [42,43]. To illustrate, redundant angiotensin II, one of the main hormones in the system, increased oxidative stress in microglia and accelerated the apoptosis of dopaminergic neurons [44]. Crucially, modulating the RAAS was shown to alleviate oxidative and ER stress and improve mitochondrial functioning [42,45].

In light of our previous observations and the functional overlap between WFS1 and the RAAS, we wanted to assess whether the RAAS is also altered in the central nervous system (CNS) of WS rats. The brain stem and hippocampus include some of the most notably affected regions in WS [46,47,48]. WFS1 is also highly abundant in these regions, predominating in the CA1 region of the hippocampus and in the brain stem nuclei [5,49].

Accordingly, for the current study, we used hippocampi and brain stem tissue collected as part of our previous long-term treatment study, wherein aged WS rats (9 months) were administered LIR and 7,8-dihydroxyflavone (7,8-DHF, an in vivo brain-derived neurotrophic factor, BDNF, mimetic) for 3.5 months. There, we showed that all treatment modalities (LIR only, 7,8-DHF only or combination) prevented lateral ventricle enlargement, reduced neuroinflammation, delayed optic nerve atrophy and improved visual acuity and learning in WS rats [26]. Therefore, we were additionally interested in evaluating the effect of these drugs on RAAS gene expression. Further, in order to control for stress induced by chronic experimental manipulations, treatment-naïve rats taken directly from their home cages were included as an experimental group.

## 2. Materials and Methods

### 2.1. Animals

For this study, outbred male CD^®^ (Sprague-Dawley) IGS homozygous *Wfs1*-deficient (*Wfs1*-ex5-KO232) rats and their wild-type (WT) littermates (as controls) were used; outbred animals were selected as these are more representative of population-level heterogeneity. *Wfs1*-ex5-KO232 mutants have previously been extensively characterized [50]. Breeding and genotyping were executed at the Laboratory Animal Centre at the University of Tartu. Animals were housed in groups of 4 under a 12 h light/dark cycle (lights on at 7 a.m.) with unlimited access to food (Sniff universal mouse and rat maintenance diet, Ssniff #V1534, ssniff Spezialdiäten, Germany) and water. All experimental protocols were approved by the Estonian Project Authorization Committee for Animal Experiments (No 155, 6 January 2020), and all experiments were performed in accordance with the European Communities Directive of September 2010 (2010/63/EU). The study was carried out in compliance with the ARRIVE guidelines.

### 2.2. Treatment and Sample Collection

Nine-month-old animals were randomly allocated to the following treatment groups: liraglutide (LIR, *n* = 5–7), 7,8-dihydroxyflavone (7,8-DHF, *n* = 5–7), liraglutide + 7,8-dihydroxyflavone (LIR + 7,8-DHF, *n* = 6–8) or control (vehicle) group (VEH, *n* = 5–7). LIR (Novo Nordisk, Denmark) was prepared in 0.9% saline; 7,8-DHF (#D1916, Tokyo Chemical Industry CO., Ltd., Japan) was first dissolved in 100% dimethyl sulfoxide (DMSO) to 400 mg/mL and further diluted 1:20 with a polyethylene glycol-300 (PEG-300)/PBS mix (1:1), resulting in a final solution of 20 mg/mL 7,8-DHF in 5% DMSO/47.5% PEG-300/47.5% PBS. The animals received a daily subcutaneous dose of LIR (0.4 mg/kg), 7,8-DHF (5 mg/kg), LIR + 7,8-DHF or the corresponding vehicle (1 mL/kg for 0.9% saline or 0.25 mL/kg for 5% DMSO/47.5% PEG-300/47.5% PBS) for 3.5 consecutive months [26]. All drug injections were performed between 8 a.m. and 11 a.m.

Of note, the animals also underwent a battery of other experimental manipulations over the study period, including routine blood sugar measurements, visual acuity measurements, cataract scoring, Morris water maze and MRI imaging under isoflurane anesthesia [26].

In order to control for the effect of repeated experimental manipulations, 12.5–13-month-old naïve WS rats and their WT littermates (*n* = 8, both groups) were used. These animals were not subjected to any treatment or manipulation and were directly euthanized from their home cages.

Both treated (within 24 h following the last injection) and naïve animals (taken directly from their home cages for downstream analyses and hereafter referred to as “treatment-naïve”) were sacrificed by decapitation. The brains were removed, and the hippocampi and brain stems were dissected, immediately washed with 0.9% saline and snap frozen in liquid nitrogen. Tissue samples were stored at −80 °C for further analysis.

### 2.3. Sample Preparation and Gene Expression Analyses

Hippocampi and brain stems were homogenized (Precellys lysing Kit CK14 + Precellys homogenizer (Bertin Instruments, Montigny-le-Bretonneux, France)), and total RNA from tissue lysates was isolated using Direct-zol RNA MiniPrep (Zymo Research, Irvine, CA, USA) according to the manufacturers’ protocol. Total RNA (500 ng) was reverse-transcribed to cDNA using random hexamers and SuperScript™ III Reverse Transcriptase (Invitrogen, Carlsbad, CA, USA).

qPCR was performed on the QuantStudio 12K Flex Real-Time PCR System (Applied Biosystem, Waltham, MA, USA) using Taqman Gene Expression Mastermix (Thermo Fisher Scientific, Baltics, Vilnius, Lithuania) with the following TaqMan Gene Expression Assays: *Ace* (angiotensin I converting enzyme; Rn00561094_m1), *Ace2* (angiotensin I converting enzyme 2; Rn01416293_m1), *Agtr1a* (angiotensin II receptor, type 1a; Rn02758772_s1), *Agtr1b* (angiotensin II receptor, type 1b; Rn02132799_s1), *Agtr2* (angiotensin II receptor, type 2; Rn00560677_s1), *Bdkrb1* (bradykinin receptor B1; Rn02064589_s1), *Bdkrb2* (bradykinin receptor B2; Rn01430057_m1) and *Mas1* (MAS1 proto-oncogene G protein-coupled receptor; Rn00562673_s1). The expression of target genes was normalized to *Hprt1* (hypoxanthine-guanine phosphoribosyltransferase; Rn01527840_m1) as an endogenous reference control. Relative expression was quantified using the 2^−ΔCt^ method [50].

### 2.4. Statistical Analysis

Statistical analyses were performed and data visualized using the GraphPad Prism software v9 (GraphPad Software Inc., San Diego, CA, USA). The data were compared using either a (i) one-way ANOVA followed by Dunnett’s multiple comparisons test or (ii) an unpaired *t*-test. The data are presented as the mean and standard error of the mean (±SEM). A *p*-value of <0.05 was considered statistically significant.

## 3. Results

### 3.1. Agtr1a, Agtr1b, Agtr2 and Bdkrb1 Levels Are Downregulated in the Hippocampi of WS Rats Receiving Chronic Treatment

The hippocampi of WS rats were analyzed to examine whether the expression of key RAAS components was affected and whether chronic drug treatment with LIR and 7,8-DHF can exert a modulatory effect.

**Figure 1:**
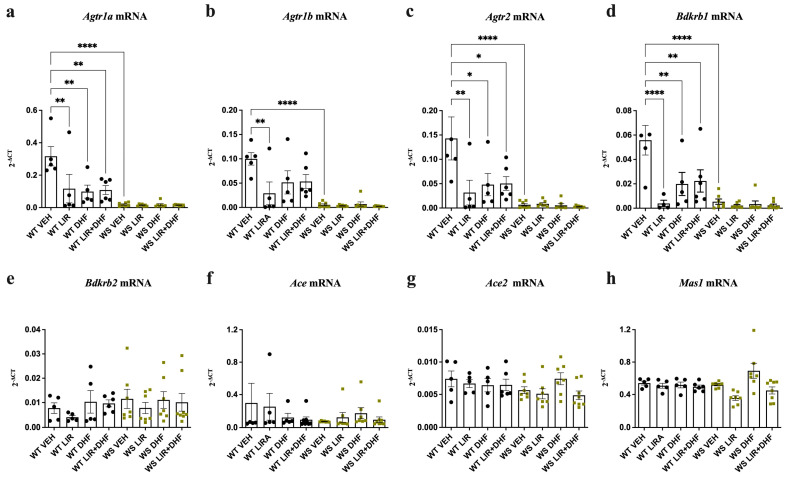
Expression of *Agtr1a*, *Agtr1b*, *Agtr2* and *Bdkrb1* was significantly downregulated in the hippocampi of chronically treated aged *Wfs1*-deficient rats. Gene expression was analyzed from the hippocampi of 12.5-month-old animals after 3.5 months of treatment with liraglutide (LIR), 7,8-dihydroxyflavone (DHF), liraglutide + 7,8-dihydroxyflavone (LIR + DHF) or vehicle (VEH). Relative gene expression levels of (**a**) *Agtr1a*, (**b**) *Agtr1b*, (**c**) *Agtr2*, (**d**) *Bdkrb1*, (**e**) *Bdkrb2*, (**f**) *Ace*, (**g**) *Ace2* and (**h**) *Mas1* (presented as 2^−ΔCT^ relative to the housekeeper *Hprt*). Statistical significance was determined using one-way ANOVA followed by Dunnett’s multiple comparisons test * *p* < 0.05; ** *p* < 0.01; **** *p* < 0.0001. The data are presented as mean ± SEM, *n*  =  5–8 per group.

First, hippocampal levels of *Agtr1a, Agtr1b, Agtr2* and *Bdkrb1* were significantly downregulated in vehicle-treated WS rats relative to their vehicle-treated WT littermates (Figure 1a–d) (*p* < 0.0001). These alterations were conserved in WS rats across all treatment groups, indicating that none of the administered drugs (LIR only, 7,8-DHF only or combination) were able to modulate this downregulation. In contrast, a treatment-induced effect was evident in WT animals; *Agtr1a, Agtr1b, Agtr2* and *Bdkrb1* were significantly downregulated across all treatment groups relative to the vehicle group (Figure 1a–d) (*p* < 0.05). Finally, no significant treatment- or genotype-driven differences were observed for *Bdkrb2*, *Ace*, *Ace2* and *Mas1* expression (Figure 1e–h).

In summary, hippocampal RAAS component expression significantly differed between WS rats and their WT littermates. Surprisingly, chronic drug treatment was unable to influence this difference, although it induced changes in the WT animals.

### 3.2. RAAS Component Expression Was Unchanged in the Brain Stems of WS Rats Receiving Chronic Treatment

**Figure 2:**
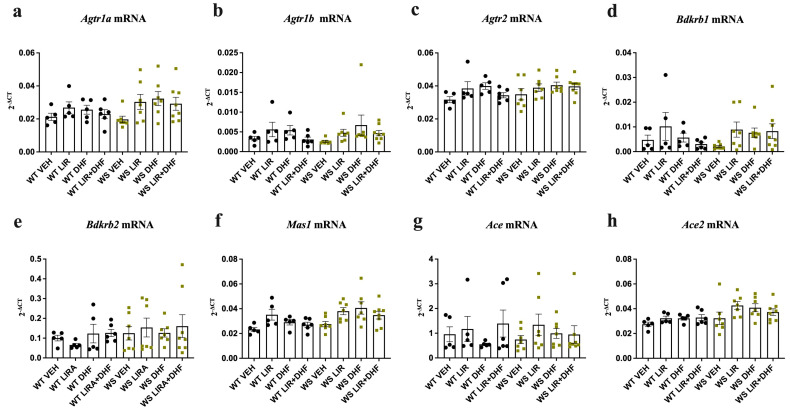
No significant between-genotype or between-treatment group differences were noted in the brain stems of chronically treated aged *Wfs1*-deficient rats. Gene expression was analyzed from the brain stems of 12.5-month-old animals after 3.5 months of treatment with liraglutide (LIR), 7,8-dihydroxyflavone (DHF), liraglutide + 7,8-dihydroxyflavone (LIR + DHF) or vehicle (VEH). Relative gene expression levels of (**a**) *Agtr1a*, (**b**) *Agtr1b*, (**c**) *Agtr2*, (**d**) *Bdkrb1*, (**e**) *Bdkrb2*, (**f**) *Ace*, (**g**) *Ace2* and (**h**) *Mas1* (presented as 2^−ΔCT^ relative to the housekeeper *Hprt*). Statistical significance was determined using one-way ANOVA followed by Dunnett’s multiple comparisons test. The data are presented as mean ± SEM, *n*  =  5–8 per group.

Genotype- and treatment-induced differences in RAAS component expression were also examined in the brain stem. However, in contrast to the observations in the hippocampi, no significant differences for any of the target genes were noted in either between-genotype or between-treatment group comparisons (Figure 2).

Taken together, and in agreement with previous observations in the heart and lungs [32], *Agtr1a*, *Agtr1b*, *Agtr2* and *Bdkrb1* gene expression was substantially downregulated in the hippocampi but not in the brainstems of WS rats relative to their WT littermates exposed to long-lasting treatment. Chronic administration of LIR, 7,8-DHF or their combination induced changes in the hippocampal expression of WT animals but had no significant effect on the expression in the brain stems of either genotype (Figure 1 vs. Figure 2). This suggests that alterations in key RAAS components may be brain region specific.

### 3.3. Ace, Ace2 and Mas1 Were Significantly Downregulated in the Hippocampi of Treatment-Naïve WS Rats

Several neuropsychiatric complications, including increased anxiety and depression, have been reported in both WS patients and animal models [51]. Moreover, both preclinical and clinical studies have demonstrated a link between RAAS alterations and these complications (for a comprehensive review, see [52]). In lieu of this, it was speculated that chronic treatment- and handling-induced stress may underlie the finding of administered treatments being unable to modulate the downregulated hippocampal levels of *Agtr1a*, *Agtr1b*, *Agtr2* and *Bdkrb1* in vehicle-treated WS rats. It was further hypothesized that fully functional WFS1 is necessary for proper functioning of the RAAS, particularly its compensatory axis, during chronic stress. To investigate this, RAAS component expression was analyzed in age-matched treatment-naïve WS and WT rats taken directly from their home cages.

**Figure 3:**
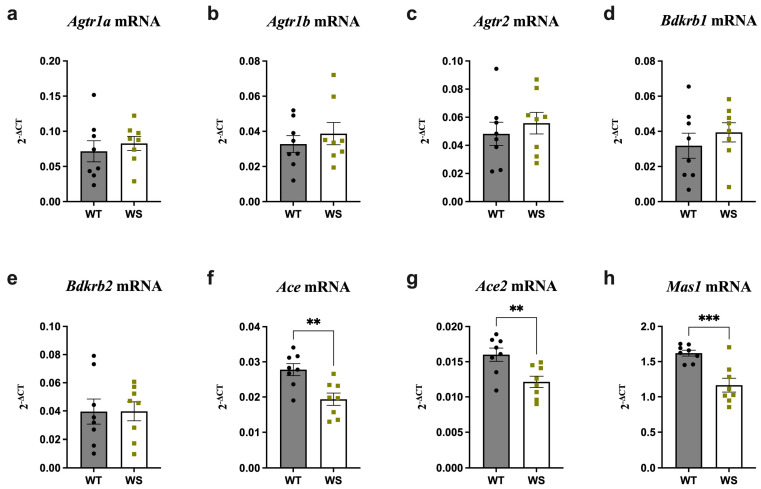
Expression of *Ace, Ace2* and *Mas1* was substantially downregulated in the hippocampi of treatment-naïve aged *Wfs1*-deficient rats. Gene expression was analyzed from the hippocampi of 12.5–13-month-old animals taken directly from their home cages. Relative gene expression levels of (**a**) *Agtr1a*, (**b**) *Agtr1b*, (**c**) *Agtr2*, (**d**) *Bdkrb1*, (**e**) *Bdkrb2*, (**f**) *Ace*, (**g**) *Ace2* and (**h**) *Mas1* (presented as 2^−ΔCT^ relative to the housekeeper *Hprt*). Statistical significance was determined using an unpaired *t*-test; ** *p* < 0.01; *** *p* < 0.001. The data are presented as mean ± SEM, *n*  =  8 per group.

Indeed, hippocampal RAAS expression in treatment-naïve rats significantly differed relative to their treated counterparts. More specifically, no differences in hippocampal *Agtr1a*, *Agtr1b*, *Agtr2* and *Bdkrb1* expression were noted between treatment-naïve WT and WS rats, in contrast to the finding of these being significantly downregulated in vehicle-treated WS rats. Rather, treatment-naïve WS rats had slightly elevated levels relative to their WT littermates (Figure 3a–d vs. Figure 1a–d). Treatment-naïve WS rats also displayed significantly downregulated *Ace*, *Ace2* and *Mas1* levels relative to their treatment-naïve WT littermates (Figure 3f–h) (*p* < 0.01).

To summarize, hippocampal RAAS expression differed considerably between treated (manipulated) and treatment-naïve (non-manipulated) WS and WT animals, indicating a potential interplay between *Wfs1* deficiency and chronic (prolonged treatment- and experiment-induced) stress in RAAS regulation.

### 3.4. Ace Was Significantly Upregulated and Agtr2 Downregulated in the Brain Stems of Treatment-Naïve WS Rats

The analysis was extended to the brain stems to examine whether RAAS alterations in treatment-naïve rats displayed the same regional specificity as in treated rats.

**Figure 4:**
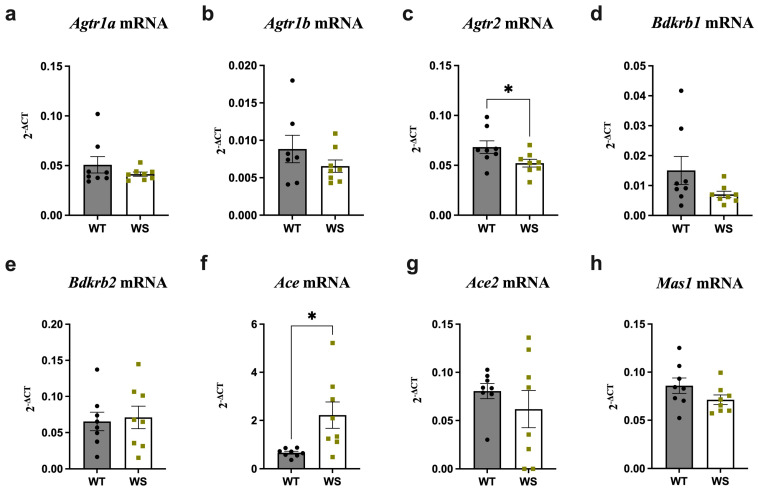
Significant upregulation and downregulation of *Ace* and *Agtr2*, respectively, was noted in the brain stems of treatment-naïve aged *Wfs1*-deficient rats. Gene expression was analyzed from the brain stems of 12.5–13-month-old animals taken directly from their home cages. Relative gene expression levels of (**a**) *Agtr1a*, (**b**) *Agtr1b*, (**c**) *Agtr2*, (**d**) *Bdkrb1*, (**e**) *Bdkrb2*, (**f**) *Ace*, (**g**) *Ace2* and (**h**) *Mas1*. Gene expression level is presented as 2^−ΔCT^ relative to the housekeeper *Hprt*. Statistical significance was determined using an unpaired *t*-test; * *p* < 0.05 The data are presented as mean ± SEM, *n*  =  8 per group.

Indeed, increased *Ace* and decreased *Agtr2* expression was seen in the brain stems of treatment-naïve WS relative to WT rats (Figure 4g,c) (*p* < 0.05). Additionally, a slight, albeit insignificant, downregulation, was observed for *Agtr1a*, *Agtr1b* and *Bdkrb1* expression in WS animals (Figure 4a,b,d). Finally, and as observed in the hippocampus, *Mas1* and *Ace2* expression was also slightly—although not significantly—decreased in WS rats (Figure 4f,h).

Altogether, region-specific differences in treatment-naïve rats were not as pronounced as those observed in treated animals.

## 4. Discussion

Mutations in a gene encoding WFS1 are the underlying cause of WS. Although WS is a monogenic disorder, pathogenic mechanisms remain poorly understood. Consequently, there is no cure for WS; nevertheless, several promising candidates, including GLP-1R agonists, have been shown to mitigate disease progression. Although this class of drugs was originally designed for the treatment of diabetes, it has demonstrated profound neuroprotective effects in preclinical models of several neurodegenerative conditions, including Alzheimer’s Disease [53], Parkinson’s Disease [54] and stroke [55].

While the functions of WFS1 remain to be fully understood, our recent study indicated a role in the modulation of the RAAS, as *Wfs1* deficiency induced profound alterations in RAAS components both in vivo and in vitro [32]. Thus, the present study sought to examine (1) the expression of key RAAS components in neural tissues from WS rats and (2) whether any observed alterations can be influenced by LIR (GLP1-R agonist) and 7,8-DHF treatment, both of which have previously demonstrated neuroprotective effects in a rat model of WS [26].

Alterations in hippocampal RAAS component expression in WS animals exposed to prolonged experimental stress were similar to those previously observed in heart, lung and primary cortical neuron cultures [32]; *Agtr2* and *Bdkrb1* levels were significantly downregulated relative to WT animals. In addition, the levels of the AGTR1 genes *Agtr1a* and *Agtr1b* were also substantially decreased. The protective functions of AGTR2 have been well established; its stimulation exerts both anti-inflammatory and anti-fibrotic effects and can promote axonal regeneration [56]. In the CNS, AGTR2 activation can induce transactivation of the brain-derived neurotrophic factor (BDNF) receptor tropomyosin receptor kinase B (TrkB), thereby facilitating BDNF/TrkB-mediated signaling. BDNF/TrkB signal transduction can activate several downstream pathways that promote cell proliferation, survival and plasticity. Disruptions in the BDNF/TRKB axis have been implicated in several neuropsychiatric conditions [57].

Both trauma and inflammation have been shown to activate BDKRB1 [58], which subsequently exerts neuroprotective effects by mediating Ca^2+^-dependent bradykinin-induced microglial migration [59]. Taken together, the loss of functional WFS1 may cause disturbances in AGTR2- and BDKRB1-mediated signaling and impair their neuroprotective effects, including cell regeneration, ER stress and inflammatory responses, thereby ultimately exacerbating WS progression. Interestingly, none of the administered treatments were able to rescue the gene downregulation observed in the hippocampi of vehicle-treated WS rats. Conversely, and surprisingly, expression levels were downregulated in WT rats across all treatment groups relative to the vehicle-treated WT rats. We speculate that this phenomenon may result, at least in part, because functional WFS1 is required for these drugs to modulate the RAAS under conditions of prolonged stress caused by long-term experimental manipulation. Additionally, there is a possibility that in WT animals, the neuroprotective potential of these drugs diminishes the need for RAAS engagement, even under chronic stress conditions. Curiously, no significant changes in the RAAS were observed in the brain stems for both between-genotype and between-treatment group comparisons in the treated rats. However, this may indicate that the interplay between WFS1 and the RAAS is influenced by time, region and environmental conditions.

Micro-RAASs can be modulated pharmacologically via cognitive processes, such as learning, as well as by chronic stress [57,60]. This is relevant, since the tissues used in the present study were collected as part of a previous study where animals continuously (3.5 months) underwent several procedures, including drug administration, vision and hearing tests and MRI-based imaging, which undoubtedly induced chronic stress [26]. Considering this and our observation that none of the treatments were able to “normalize” the alterations observed in vehicle-treated WS animals, we speculate that functional WFS1 is required to support the hippocampal RAAS response to chronic stress. Thus, treatment-naïve rats were studied to control for the effects of treatment-induced stress. Indeed, we found that these rats had decreased hippocampal expression of *Ace*, *Ace2* and *Mas1*, but no changes were observed for *Agtr2*, *Agtr1a*, *Agtr1b* and *Bdkrb1*, as seen in treated animals. Furthermore, as in treated animals, RAAS alterations in treatment-naïve rats displayed regional specificity when comparing the hippocampi and brain stems.

Decreased levels of hippocampal *Ace* and *Ace2* in treatment-naïve WS rats may indicate disturbances in angiotensin processing and consequently compromised AGTR1-, AGTR2- and MAS1-facilitated signaling. Furthermore, changes in neural ACE and ACE2 activity increase neuronal vulnerability to ER stress and inflammation and facilitate the accumulation of bradykinin and proteins such as tau and amyloid-β, all of which are implicated in neurodegenerative pathologies [61,62,63,64]. Similarly, ACE inhibition can delay neurodegeneration via the retardation of tau hyperphosphorylation [65], while ACE2 and AGTR2 activation can protect against cognitive impairments [66]. ACE inhibitors may improve cognitive functioning, including learning and memory, by activating the Ang-(1–7)/Mas axis [67]. Interestingly, a recent study found that WFS1-positive neurons in the entorhinal cortex express tau and mediate its shift to the hippocampal CA1 pyramidal cells, leading to a decline in learning and memory [68,69]. Increased vulnerability to tau pathology in WS indicates that, similarly to ACE, WFS1 interacts with tau and mediates its effects [70]. To conclude, the modulation of RAAS components can influence cognitive processes.

Present and previous findings indicate that the loss of functional WFS1 might disturb RAAS functioning, as evidenced by alterations in its key components, both peripherally and in the nervous system [32]. These disturbances may consequently augment oxidative stress, impair inflammatory responses and Ca^2+^ homeostasis, affect cognition and contribute to the development of neuropsychiatric complications. An interaction between WFS1 and key RAAS components is further supported by their co-expression in various tissues, including the brain, retina, pancreas, heart and lungs (in humans [71]), and their somewhat overlapping roles. WFS1 may potentially affect RAAS regulation under stressful conditions and facilitate the functioning of the system’s stress-response compensatory axis; disturbances in this axis, as seen here, could therefore exacerbate the course of WS disease.

GLP1-R activation can alleviate ER stress and improve cell survival and mitochondrial function via several pathways [72,73], including the ACE2-mediated RAAS compensatory axis: Ace2/Ang-(1–7)/Mas1/Agtr2. This axis supports cellular function and survival via the induction of a strong ER stress response and anti-inflammatory and regenerative pathways [74,75]. Our previous study demonstrated that LIR treatment, in addition to exerting neuroprotective effects and supporting cognitive function, could modulate the RAAS in peripheral organs [32]. Accordingly, we hypothesized that these positive effects may lie downstream of neural RAAS modulation. Here, we found that differentially expressed RAAS genes in the neural tissues of WS animals were not normalized by LIR treatment, suggesting that LIR’s efficacy derives from the modulation of other signaling and/or homeostatic pathways. In the brain, GLP-1Rs are abundant in pyramidal neurons, and their expression is induced by injury in astrocytes and GABAergic interneurons [76,77,78]. Moreover, GLP-1R agonists have been shown to abate microglial activation in vivo in WS rats [25] and increase GABAergic neurotransmission in different disease conditions, including ischemia [78,79]. Interestingly, GABA receptor activation could significantly delay neuronal death in ischemia-induced injury [80]. Accordingly, while the exact mechanisms underlying LIR’s neuroprotective effects in WS remain to be fully elucidated, they may include ameliorating reactive gliosis by modulating GABAergic signalling and/or augmenting ACE2 activity [33].

## 5. Conclusions

To summarize, the present study showed that the neural RAAS is altered in WS, as evidenced by the substantial changes in the expression of two key receptors, *Agtr2* and *Bdkrb1*. However, those alterations are not conserved across different regions, potentially owing to the differential regional, environmental and temporal modulation of the RAAS across the WS disease course.

Crucially, we showed that those changes vary depending on whether or not animals are exposed to a prolonged stressful environment (long-term animal experimentation), indicating a role played by chronic stress. Stress may further compound the effects of *Wfs1* deficiency on RAAS function, and a compromised compensatory axis could ultimately exacerbate the disease process. These results emphasize once more that experimental design and environment can affect gene expression, and that there is a strong need to control for procedural stress and include treatment-naïve animals within experimental paradigms. Finally, we showed that none of the alterations observed in vehicle-treated WS rats were amenable to pharmacological modulation, despite animals experiencing symptomatic improvement in our previous study [26]. This suggests that the neuroprotective effects of these drugs in WS are likely mediated independently of the RAAS.

## 6. Limitations of the Study

The present study is not without its limitations; alterations were only described at the transcriptomic level, and since protein-level changes were beyond the scope of this study, as it was exploratory, we recommend that future studies address this. Furthermore, experimental tissue samples were harvested from aged rats that had already developed substantial neurological symptoms, including impaired cognitive function and hippocampal lateral ventricle enlargement. Future studies may also consider investigating transcriptomic changes within specific neuronal populations, especially in regions as diverse as the brain stem. Examining the temporal development of RAAS disruptions across the WS disease course also warrants investigation. Finally, the chronic stress conditions described in this study resulted inadvertently from prolonged experimental handling. Additional analyses using classical stress paradigms should be performed to verify the results reported here.

## Data Availability

The datasets generated and analyzed during this study are available from the corresponding authors on reasonable request.

## Appendix A

List of Abbreviations (in alphabetical order)

7,8-DHF	7,8-dihydroxyflavone
Ace	Angiotensin I converting enzyme
Ace2	Angiotensin I converting enzyme 2
Agtr1a	Angiotensin II receptor type 1a
Agtr1b	Angiotensin II receptor type 1b
Agtr2	Angiotensin II receptor type 2
ARRIVE	Animal Research: Reporting of In Vivo Experiments
Bdkrb1	Bradykinin receptor B1
Bdkrb2	Bradykinin receptor B2
BDNF	Brain-derived neurotrophic factor
CNS	Central nervous system
DMSO	Dimethyl sulfoxide
ER	Endoplasmic reticulum
GABA	Gamma-aminobutyric acid
GLP-1R	Glucagon-like peptide 1 receptor
Hprt1	Hypoxanthine-guanine phosphoribosyltransferase
LIR	Liraglutide
MAM	Mitochondria-associated ER membraane
Mas1	MAS1 proto-oncogene, G protein-coupled receptor
PBS	Phosphate-buffered saline
PEG-300	Polyethylene glycol-300
RAAS	Renin-angiotensin-aldosterone system
SEM	Standard error of the mean
TrkB	Tropomyosin receptor kinase B
VEH	Vehicle
WFS1	Wolframin/Wolfram Syndrome 1
WS	Wolfram Syndrome
WT	Wild-type
